# Changes in cerebral cortex activation during upright standing tasks in individuals with chronic neck pain: an fNIRS study

**DOI:** 10.3389/fneur.2025.1531314

**Published:** 2025-02-28

**Authors:** Chongwu Xiao, Qianfei Liang, Yugang Yang, Mingyu Mo, Weixiong Li, Huade Chen, Yaobin Long, Jinjun Huang

**Affiliations:** ^1^Department of Rehabilitation Medicine, The Second Affiliated Hospital of Guangxi Medical University, Nanning, Guangxi, China; ^2^Department of Rehabilitation Medicine, Guiping People’s Hospital, Guiping, Guangxi, China

**Keywords:** chronic neck pain, functional near-infrared spectroscopy, postural control, balance, standing

## Abstract

**Introduction:**

Studies show that individuals with chronic neck pain (CNP) exhibit postural control deficits, potentially contributing to persistent and recurrent pain. However, the neural mechanisms underlying these deficits in CNP remain unexplored despite their importance for developing effective rehabilitation strategies. Therefore, this study aimed to investigate the neural activity during postural control using functional near-infrared spectroscopy (fNIRS), providing insights into the central mechanism underlying postural control deficits in individuals with CNP.

**Methods:**

In this cross-sectional study, 10 individuals with CNP (CNP group) and 10 healthy controls (HC group) were assessed under three conditions: Task 1, standing on a force plate with eyes open and both feet; Task 2, standing on a force plate with eyes closed and both feet; Task 3, standing on a force plate with eyes closed and one foot. Cerebral cortex hemodynamic reactions, including bilateral prefrontal cortex (PFC), dorsolateral prefrontal cortex (DLPFC), pre-motor cortex and supplementary motor area (PMC/SMA), primary motor cortex (M1), and primary somatosensory cortex (S1) were measured using fNIRS. Balance parameters, including the sway area, total sway length, mean velocity, and center of pressure (COP) amplitude in the anterior–posterior (AP) and medial-lateral (ML) directions, were measured using a force plate.

**Results:**

In Tasks 1 and 2, no differences were observed between both groups in balance parameters. However, the CNP group exhibited significantly higher activation in the left PMC/SMA (*F* = 4.788, *p* = 0.042) and M1 (*F* = 9.598, *p* = 0.006) in Task 1 and lower activation in the left (*F* = 4.952, *p* = 0.039) and right (*F* = 6.035, *p* = 0.024) PFC in Task 2 compared to that of the HC group. In Task 3, the CNP group exhibited a significantly larger COP amplitude in the AP direction (*F* = 7.057, *p* = 0.016) compared to that of the HC group. Additionally, activation in the right M1 (*F* = 7.873, *p* = 0.012) was significantly higher than in the HC group. Correlation analysis in Task 3 revealed stronger associations between the parameters in the CNP group.

**Conclusion:**

Our findings suggest that individuals with CNP exhibit distinct patterns of cerebral cortex activities and postural control deficits. The PFC, M1, and PMC/SMA were involved in maintaining upright standing balance, and cerebral cortex changes associated with upright standing balance provide a more sensitive indicator of postural control deficits than peripheral balance parameters in individuals with CNP.

## 1 Introduction

Chronic neck pain (CNP) has become a widespread global health issue. Global data indicated that approximately 203 million people were affected by neck pain in 2020, and this number is projected to rise to 269 million by 2050 (1). CNP affects individuals and society, contributing to a substantial economic burden through healthcare costs, decreased productivity, and low quality of life (2–4). The burden of this condition is likely to continue increasing in the future, particularly among aging populations and individuals in high-risk occupations (5). The etiology of CNP is complex, with evidence suggesting that changes in the structure and function of neck musculature may play a key role in its development (6–8). These changes may impair posture control—an essential function that primarily relies on the integration of sensory inputs, neuromuscular regulation, and coordinated muscular responses (9). Moreover, research highlights a relationship between CNP and impaired postural balance, indicating a potential bidirectional interaction where each condition may exacerbate the other (10). Exploring the cortical activation and further understanding the mechanisms underlying impaired postural control in individuals with CNP is essential for developing targeted rehabilitation strategies that address pain management and functional stability.

Postural control involves involuntary and voluntary components. Voluntary postural control specifically refers to the ability to maintain stability and spatial orientation during self-initiated movements (11). Voluntary postural control is essential for performing daily life activities, as it enables individuals to coordinate movement while maintaining stability. Research focusing on voluntary postural control in the context of real-life activities is particularly valuable, as it offers insights directly relevant to the functional tasks individuals encounter in daily life. The Balance Evaluation System Test (BESTest) is a widely used tool for assessing voluntary postural control abilities. Common tasks in the BESTest include standing with the feet together and eyes open, standing with the feet together and eyes closed, and single-leg stance. These tasks provide valuable insight into postural control under varying sensory conditions (12). Studies show that individuals with impaired proprioception can compensate by relying on visual feedback, effectively stabilizing their posture despite deficits in proprioceptive input (13, 14). Therefore, assessing postural control in conditions where vision is removed, such as during eyes-closed tasks, may more effectively isolate proprioceptive deficits and elucidate underlying issues in postural control mechanisms.

Neuroimaging studies indicate that both direct and indirect motor networks are essential for postural control in CNP (15, 16). The direct motor network, including the primary motor cortex (M1) and cerebellum, is primarily responsible for motor execution. In contrast, the indirect motor network, involving regions such as the prefrontal cortex (PFC) and the supplementary motor area, plays a key role in motor planning and coordination (17). Previous research utilizing functional magnetic resonance imaging (fMRI) has explored functional and structural changes in the brains of patients with CNP, revealing potential links between postural control deficits and altered brain function (18, 19). One study indicates decreased gray matter volume in the right mid-cingulate cortex, right superior temporal gyrus, and right precuneus in people with CNP, alongside reduced functional connectivity between the right precuneus and bilateral medial PFC (18). Another study reports enhanced functional coupling between the left amygdala and frontal operculum in individuals with CNP at rest (19), while another indicates altered network properties in the posterior cingulate cortex, amygdala, and globus pallidus (20). These findings suggest a reorganization of brain networks, highlighting the importance of targeted brain-based interventions in rehabilitating CNP. Functional near-infrared spectroscopy (fNIRS) has also been employed in chronic pain research, revealing that postural control during an upright stance is maintained by the pre-motor cortex and supplementary motor area (PMC/SMA) alongside the dorsolateral prefrontal cortex (DLPFC), in patients with chronic low back pain (21). However, studies specifically linking fNIRS cerebral cortex activation to postural control deficits in CNP remain limited, highlighting the need for further exploration in this area.

fNIRS is a noninvasive optical neuroimaging technique that relies on neurovascular coupling and spectroscopy principles, offering a unique observational tool for basic neuroscience and clinical applications (22). Compared to traditional neuroimaging methods, fNIRS provides unique advantages for studying postural control. First, its high flexibility allows data collection without requiring a stationary position, making it particularly well-suited for dynamic tasks. Second, fNIRS can effectively detect and correct motion artifacts, ensuring high-quality signals even during movement. Finally, with its high temporal and spatial resolution, fNIRS enables real-time monitoring of oxygenation changes within the cerebral cortex, providing valuable insights into functional brain activity (23, 24). These advantages make fNIRS highly suitable for investigating neural activity involved in postural control (25). Despite these advantages, studies employing fNIRS to assess postural control ability during upright standing tasks in individuals with CNP remain lacking.

Therefore, this study aimed to investigate cerebral cortex activation in individuals with CNP using fNIRS while performing various upright standing tasks on a force plate. We hypothesized that individuals with CNP would exhibit postural control deficits, characterized by increased cerebral cortex activation compared to that of healthy individuals and that balance parameters would correlate with cerebral cortex activation levels. The results would contribute to the understanding of neural activity during postural control in individuals with CNP, offering valuable insights for further exploration of the central mechanism underlying postural control deficits.

## 2 Materials and methods

### 2.1 Participants

The participants were recruited from the Department of Rehabilitation at the Second Affiliated Hospital of Guangxi Medical University and nearby communities. Overall, 10 individuals with CNP (CNP group) and 10 healthy controls (HC group) were enrolled in this study. Approval was obtained from the Ethics Committee of the Second Affiliated Hospital of Guangxi Medical University (approval number: 2024-KY (0747)). All participants provided written informed consent in accordance with the Declaration of Helsinki.

The sample size was calculated using G*Power 3.1.9.2 (Kiel University, Kiel, Germany). Based on previous research on static standing balance function in individuals with CNP and healthy controls (26), the center of pressure (COP) amplitude in the anterior–posterior (AP) direction during eyes-closed standing was reported as 39.89 mm and 29.23 mm for individuals with CNP and healthy controls, with standard deviations (SD) of 9.47 mm and 8.27 mm, respectively. The calculated effect size was 1.20. With an *α*-level set at 0.05, a power of 0.8, and an allocation ratio of 1:1 between both groups, the final sample size was determined to be 20 participants, with 10 in each group.

The inclusion criteria were as follows: (a) neck pain or discomfort lasting ≥3 months; (b) a Numeric Pain Rating Scale (NPRS) score of ≥3 and a Neck Disability Index (NDI) score of ≥10; (c) right-handed; (d) aged between 18 and 75 years; and (e) able to stand independently as required for the study. The exclusion criteria were as follows: (a) use of medications that could affect postural stability (e.g., sedatives or hypnotics); (b) history of hip or knee joint replacement surgery affecting standing; (c) presence of other musculoskeletal pain that could affect balance (e.g., low back and leg pain); (d) neurological or sensory disorders that impair postural stability (e.g., diabetes, Parkinson’s disease, peripheral neuropathy, cerebellar disorders, vestibular diseases, psychiatric disorders, or visual or hearing impairments); and (e) pregnancy.

### 2.2 Clinical measurements

Demographic data, including sex, age, height, weight, Body Mass Index (BMI), years of education, and duration of pain, were primarily collected through self-report by the participants. The Berg Balance Scale (BBS) was used to assess the balance function of the participants. The scale consists of 14 items, with a total score of 56 points; higher scores indicate a better balance function. The reliability of the scale is 0.97 (27). The NPRS was used to measure pain intensity in individuals with CNP, with scores ranging from 0 to 10, where higher scores indicate greater pain severity. The intraclass correlation coefficient for the NPRS is 0.99, and the area under the curve is 0.88, making it the preferred tool for assessing pain severity (28). The NDI was used to assess the level of functional disability in individuals with CNP. The total score ranges from 0 to 50 points, with higher scores indicating greater severe functional disability. The reliability of the Chinese version of the NDI is 0.92 (29). The above measurement indices were collected before commencing the upright standing tasks, taking approximately 30 min.

### 2.3 Kinematic measurements

The balance parameters of both groups during the upright standing tasks were assessed using the AL-600 Gait and Balance Function Training and Evaluation System (Aili Intelligent Technology Co., Ltd., Hefei, China). The system includes 2,400 pressure array sensors, a high-precision sensor force plate (400 × 600 mm), a high-speed matrix acquisition circuit with a sampling frequency of 100 Hz, and software modules. The system calculates the COP trajectory and sway area based on the pressure distribution across the contact surface, and it can display the real-time position of the COP on the screen. Based on previous studies on upright standing (30, 31), the balance parameters assessed in this study included the sway area, total sway length, mean velocity, and COP amplitude in the AP and medial-lateral (ML) directions. The COP amplitude in the AP and ML directions was calculated based on the movement of the COP in the sagittal and coronal planes, respectively. The sway area represented the surface area covered by the COP during motion. The mean velocity was obtained by dividing the total displacement of the COP by the task duration. The total sway length represents the cumulative distance traveled by the COP.

### 2.4 fNIRS measurements and data processing

**Figure 1:**
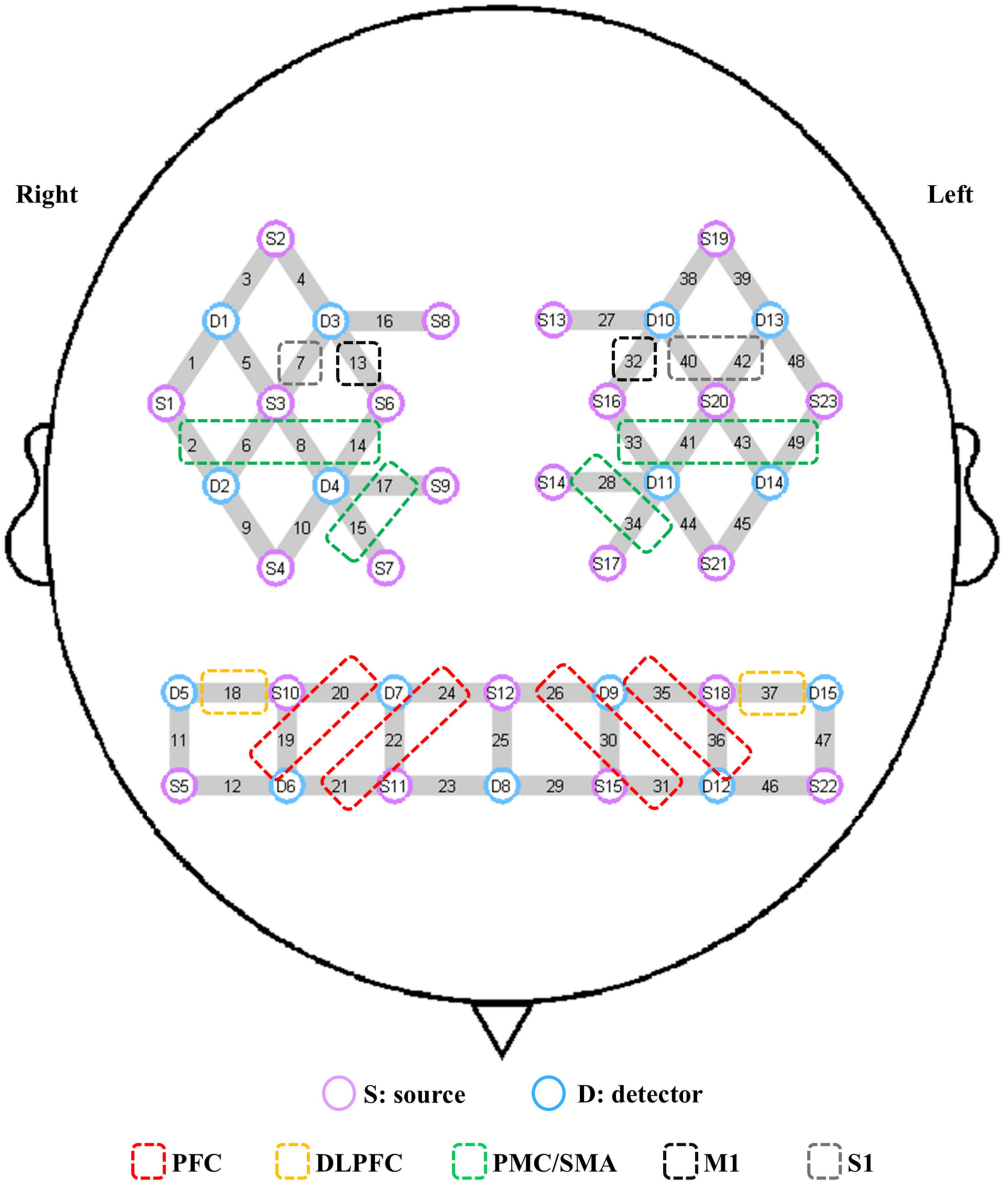
Channel composition of the ROIs. S, source; D, detector; PFC, prefrontal cortex; DLPFC, dorsolateral prefrontal cortex; PMC/SMA, pre-motor cortex and supplementary motor area; M1, primary motor cortex; S1, primary somatosensory cortex; ROI, region of interest.

In this study, a multichannel fNIRS device (NirSmart, Danyang Huichuang Medical Equipment Co., Ltd., Jiangsu, China) was used to detect changes in the concentrations of oxygenated hemoglobin (HbO) in the region of interest (ROI) during upright standing tasks. These changes reflected cerebral cortex activation. The device consists of near-infrared light sources (light-emitting diodes, LED) and avalanche photodiodes as detectors. The wavelengths were set to 730 and 850 nm, with data sampled at a frequency of 11 Hz. Twenty-three sources and 15 detectors were used in the experiment to create 49 measurement channels, with an average distance of 3.0 cm between the source and detector. The probe coordinates were positioned according to the international 10–20 system, then converted into MNI coordinates and projected onto the MNI standard brain template using a spatial registration approach in NirSpace (Danyang Huichuang Medical Equipment Co., Ltd., Jiangsu, China). Based on previous studies (21, 32), the ROIs in this study included the left and right PFC, DLPFC, PMC/SMA, M1, and primary somatosensory cortex (S1). The left PFC consisted of channels 26, 30, 31, 35, and 36, while the right PFC comprised channels 19, 20, 21, 22, and 24. The left and right DLPFC were covered by channels 37 and 18, respectively. The left PMC/SMA was composed of channels 28, 33, 34, 41, 43, and 49, while the right was comprised of channels 2, 6, 8, 14, 15, and 17. The left and right M1 were covered by channels 32 and 13, respectively. The left and right S1 were represented by channels 40, 42 and 13, respectively (Figure 1). Group analysis was performed based on these ROIs.

The original data collected were preprocessed using NirSpark, a MATLAB-based optical imaging software (Danyang Huichuang Medical Equipment Co., Ltd., Jiangsu, China). The specific steps were as follows: First, the raw intensity data were converted into optical density data. Second, a spline interpolation algorithm was applied to the resulting signals to correct motion artifacts by channels. The advantage of spline interpolation was that it selectively corrected only the pre-localized artifacts. Third, a bandpass filter (0.01–0.2 Hz) was applied to eliminate noise caused by physiological fluctuations, such as pulse and respiration. Finally, the modified Beer–Lambert law was used to calculate the relative changes in hemoglobin concentration, specifically in HbO and deoxygen-hemoglobin (33), with differential pathlength factor setting as 6 for each wavelength. Our study focused solely on changes in HbO concentration. We performed baseline correction on the HbO concentration during the first 5 s before the upright standing task and calculated the change in HbO concentration during the task relative to the baseline. This reflected cerebral cortex activation (34). The HbO concentrations for each block paradigm were superimposed and averaged to generate a block average result.

### 2.5 Experiment procedures

**Figure 2:**
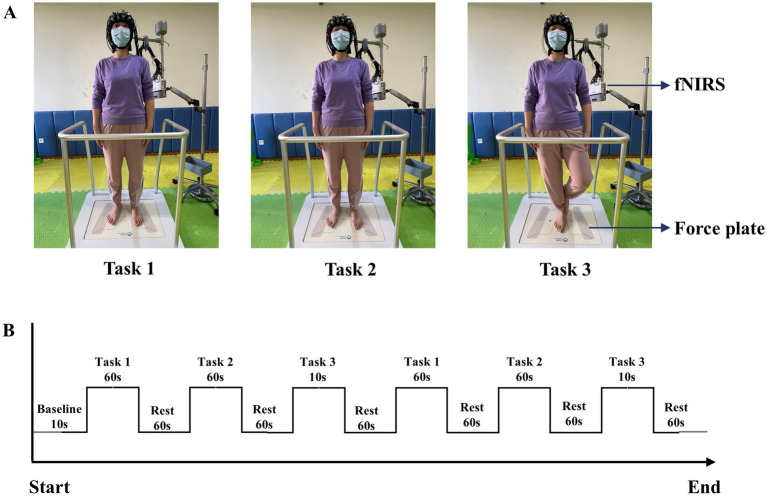
**(A)** Diagram of three upright standing tasks. **(B)** fNIRS measurement procedure. Task 1: standing on the force plate with eyes open and both feet; Task 2: standing on the force plate with eyes closed and both feet; Task 3: standing on the force plate with eyes closed and one foot.

Participants performed three upright standing tasks with varying difficulties: Task 1: standing on the force plate with eyes open and both feet; Task 2: standing on the force plate with eyes closed and both feet; Task 3: standing on the force plate with eyes closed and one foot (Figure 2A). These tasks have been employed in previous researches on upright standing balance (26, 35). Before beginning the tasks, the surrounding environment was ensured to be quiet and well-lit. The participants were instructed to remove their shoes and practice the upright standing tasks twice under the guidance of a therapist. The task order was blinded to the participants. Following that, they donned the fNIRS device and stood on the force plate. Participants maintained a natural standing posture with their arms relaxed at their sides, facing a point on the wall at eye level, approximately 1.5 m away (36), then the data of the baseline period was collected. In the experimental period, each task was standardized and repeated twice. For the relatively simple tasks (1 and 2), the duration was set to 60 s. Based on our prior experimental experience, the more challenging Task 3 was designed to ensure a higher completion rate with a 10 s duration. After each task, participants rested for 60 s to allow HbO concentration to return to baseline (Figure 2B), maintaining the same posture as the baseline period. If participants spoke, fell, or moved from the initial position during the tasks, the measurement was terminated. They were given 10 min rest before attempting the tasks again. Throughout the task, the therapist was unaware of the pain conditions of the participants. The average value from the repeated trials was taken and used for analysis.

### 2.6 Statistical analysis

Statistical analyses were conducted using IBM SPSS Statistics version 23.0 (IBM Corp., Armonk, NY, United States). Continuous variables were presented as mean ± SD, while categorical variables were expressed as frequencies and percentages. The Chi-square test was used to analyze differences in gender and occupation between the two groups. The Kolmogorov–Smirnov test was used to assess the normality of data for age, height, weight, BMI, years of education, and duration of pain. An independent *t*-test was conducted for normally distributed data, while a non-parametric test was applied to data that did not meet the normality assumption. A one-way analysis of variance (ANOVA) or a non-parametric test was used to determine significant differences in HbO concentration and kinematic parameters during the upright standing tasks between the two groups. Statistical results were adjusted for multiple comparisons across ROIs using the false discovery rate (FDR) method (37). Pearson’s correlation analysis was used to evaluate the relationship between HbO concentration and kinematic parameters when the data followed a normal distribution; otherwise, Spearman’s correlation analysis was applied. GraphPad Prism 10.0 software was utilized for chart editing. *p* < 0.05 was considered statistically significant.

## 3 Results

### 3.1 Demographic characteristics

Statistical analysis revealed no significant differences between the two groups in gender, age, height, BMI, or years of education (*p* > 0.05). Furthermore, no statistically significant differences were observed in BBS scores between the groups (*p* > 0.05). Participants in the CNP group reported an average pain duration of 49 months, with a mean NPRS score of 5.4 and an NDI score of 16.3 (Table 1).

**Table 1:** The demographic characteristics of the two groups (mean ± SD).

Variable	HC group (*n* = 10)	CNP group (*n* = 10)	*p*
Sex			
Female	8 (80%)	8 (80%)	1.000
Male	2 (20%)	2 (20%)	
Age (years)	36.20 ± 12.54	45.40 ± 11.62	0.106
Height (m)	1.62 ± 0.08	1.59 ± 0.07	0.254
Weight (kg)	60.90 ± 12.91	57.80 ± 7.22	0.516
BMI (kg/m^2^)	22.99 ± 3.68	22.99 ± 2.78	0.997
Years of education (years)	16.00 ± 1.70	15.50 ± 1.51	0.496
BBS (maximum = 56)	55.70 ± 0.68	54.80 ± 1.62	0.131
Duration of pain (months)	N/A	49.00 ± 98.91	N/A
NPRS (0–10)	N/A	5.40 ± 1.27	N/A
NDI (maximum = 50)	N/A	16.30 ± 6.95	N/A

N/A, not applicable; HC, health control; CNP, chronic neck pain; BMI, body mass index; BBS, Berg Balance Scale; NPRS, Numeric Pain Rating Scale; NDI, Neck Disability Index.

### 3.2 Comparison of upright standing balance performance between groups

A one-way ANOVA revealed no significant differences between the two groups in the six balance parameters during Task 1 and Task 2 (*p* > 0.05). However, in Task 3, the COP amplitude in the AP direction for the CNP group was significantly greater than that for the HC group (*F* = 7.057, *p* = 0.016, η^2^ = 0.282), with no significant differences observed in the other parameters (*p* > 0.05) (Table 2).

**Table 2:** Results of the one-way ANOVA for balance parameters during upright standing tasks between the groups (mean ± SD).

Variable	HC group (*n* = 10)	CNP group (*n* = 10)	*F*	*p*	η^2^
Task 1					
COP AP-Amplitude (cm)	2.159 ± 0.395	2.467 ± 0.768	1.275	0.274	0.066
COP ML-Amplitude (cm)	1.344 ± 0.369	1.661 ± 0.491	2.657	0.120	0.129
mean AP-velocity (cm/s)	1.840 ± 0.326	1.840 ± 0.233	0.000	0.100	0.000
mean ML-velocity (cm/s)	2.252 ± 0.498	2.555 ± 0.340	0.025	0.877	0.001
total sway length (cm)	206.859 ± 38.811	208.930 ± 27.096	0.019	0.893	0.001
sway area (cm^2^)	2.014 ± 0.731	2.938 ± 1.670	2.571	0.126	0.125
Task 2					
COP AP-Amplitude (cm)	3.400 ± 1.964	3.688 ± 1.289	0.450	0.703	0.008
COP ML-Amplitude (cm)	1.495 ± 0.393	2.214 ± 0.868	4.349	0.052	0.195
mean AP-velocity (cm/s)	1.995 ± 0.305	2.050 ± 0.239	0.201	0.659	0.000
mean ML-velocity (cm/s)	2.550 ± 0.494	2.600 ± 0.346	0.069	0.796	0.004
total sway length (cm)	215.242 ± 37.714	218.861 ± 26.261	0.062	0.806	0.003
sway area (cm^2^)	3.313 ± 2.514	5.709 ± 3.529	0.058	0.097	0.145
Task 3					
COP AP-Amplitude (cm)	4.785 ± 1.626	8.539 ± 4.163	7.057	**0.016**	0.282
COP ML-Amplitude (cm)	5.255 ± 5.003	9.432 ± 9.058	1.629	0.218	0.083
mean AP-velocity (cm/s)	4.755 ± 2.206	6.530 ± 2.938	2.334	0.144	0.115
mean ML-velocity (cm/s)	5.305 ± 2.446	7.275 ± 3.094	2.495	0.132	0.122
total sway length (cm)	75.978 ± 32.932	101.070 ± 41.666	2.232	0.152	0.110
sway area (cm^2^)	20.935 ± 23.448	54.223 ± 74.744	1.806	0.196	0.091

HC, health control; CNP, chronic neck pain; COP, center of pressure; AP, anterior–posterior; ML, medial-lateral; Task 1, standing on the force plate with eyes open and both feet; Task 2, standing on the force plate with eyes closed and both feet; Task 3, standing on the force plate eyes with closed and one foot. The bold values mean significant difference.

### 3.3 Results of cerebral cortex activation between groups during tasks

**Figure 3:**
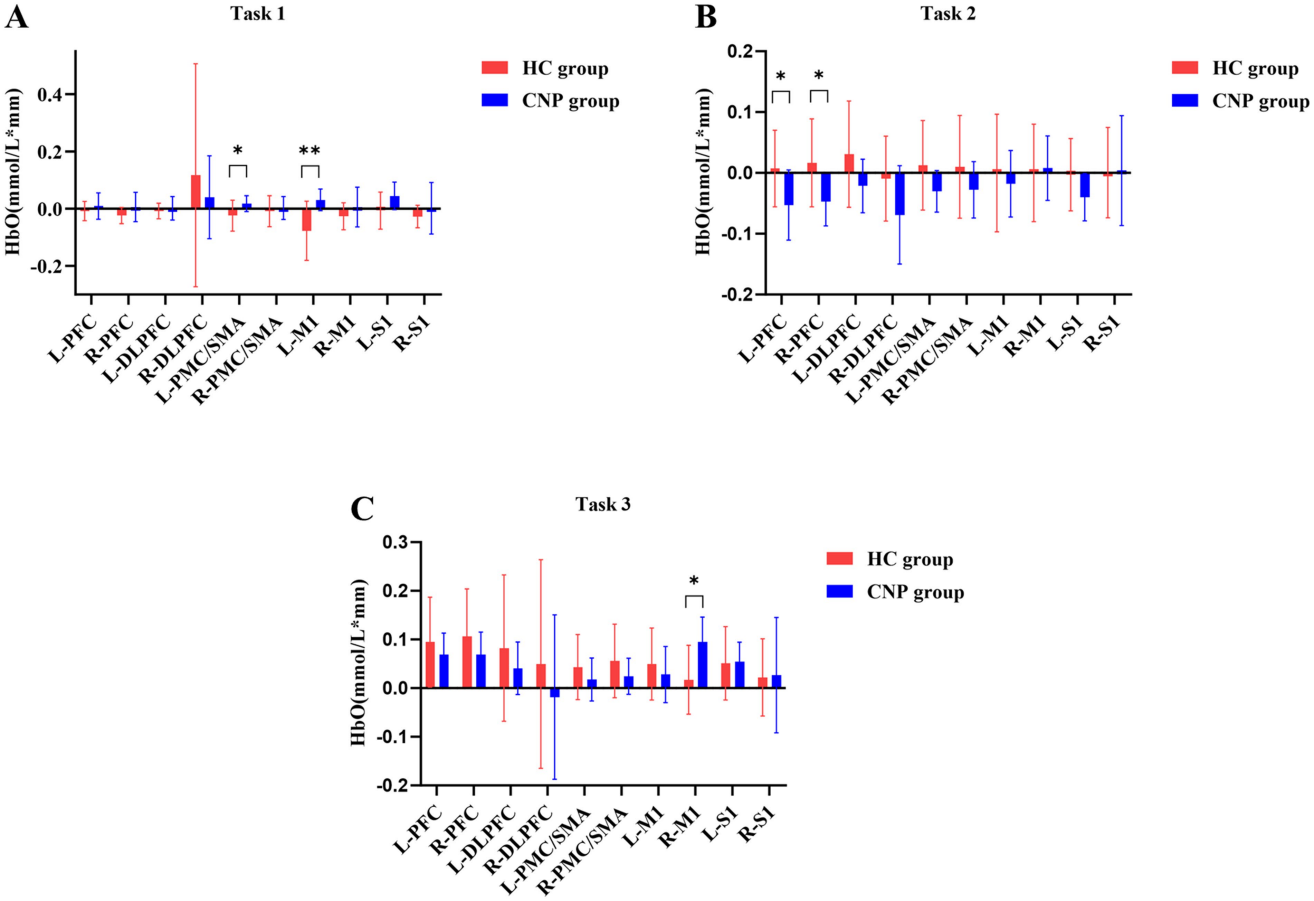
Comparison of the cerebral cortex activation during **(A)** tasks 1, **(B)** 2, and **(C)** 3 between both groups. L, left; R, right; PFC, prefrontal cortex; DLPFC, dorsolateral prefrontal cortex; PMC/SMA, pre-motor cortex and supplementary motor area; M1, primary motor cortex; S1, primary somatosensory cortex; ^*^ *p* < 0.05; ^**^ *p* < 0.01; Error bars indicate 95% confidence interval.

**Figure 4:**
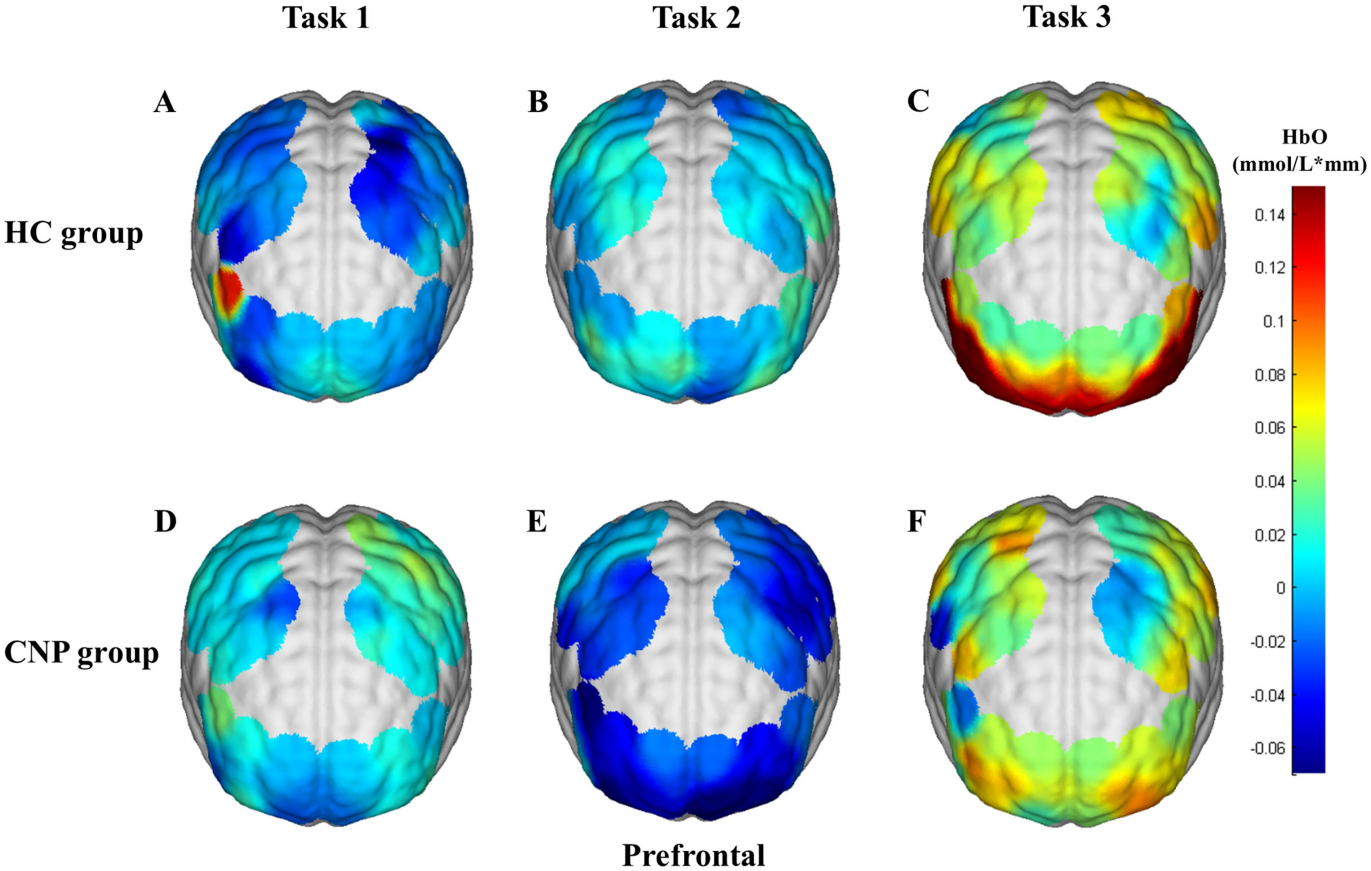
3D brain map of average cerebral cortex activation in tasks **(A)** 1, **(B)** 2, and **(C)** 3 in the HC group and tasks **(D)** 1, **(E)** 2, and **(F)** 3 in the CNP group. Red and blue represent hyperactivation and hypoactivation, respectively.

A one-way ANOVA revealed that the CNP group exhibited significantly higher cerebral cortex activation in the left PMC/SMA (*F* = 4.788, *p* = 0.042, η^2^ = 0.210) and left M1 (*F* = 9.598, *p* = 0.006, η^2^ = 0.348) than in the HC group during Task 1 (Figure 3A). During Task 2, the CNP group exhibited significantly lower cerebral cortex activation in the left PFC (*F* = 4.952, *p* = 0.039, η^2^ = 0.216) and right PFC (*F* = 6.035, *p* = 0.024, η^2^ = 0.251) than in the HC group (Figure 3B). In contrast, during Task 3, the CNP group demonstrated significantly higher cerebral cortex activation in the right M1 (*F* = 7.873, *p* = 0.012, η^2^ = 0.304) than in the HC group (Figure 3C). Figure 4 illustrates the 3D brain map depicting average cerebral cortex activation across the three tasks for both groups.

### 3.4 Association between cerebral cortex oxygenated hemoglobin and upright standing balance parameters

**Figure 5:**
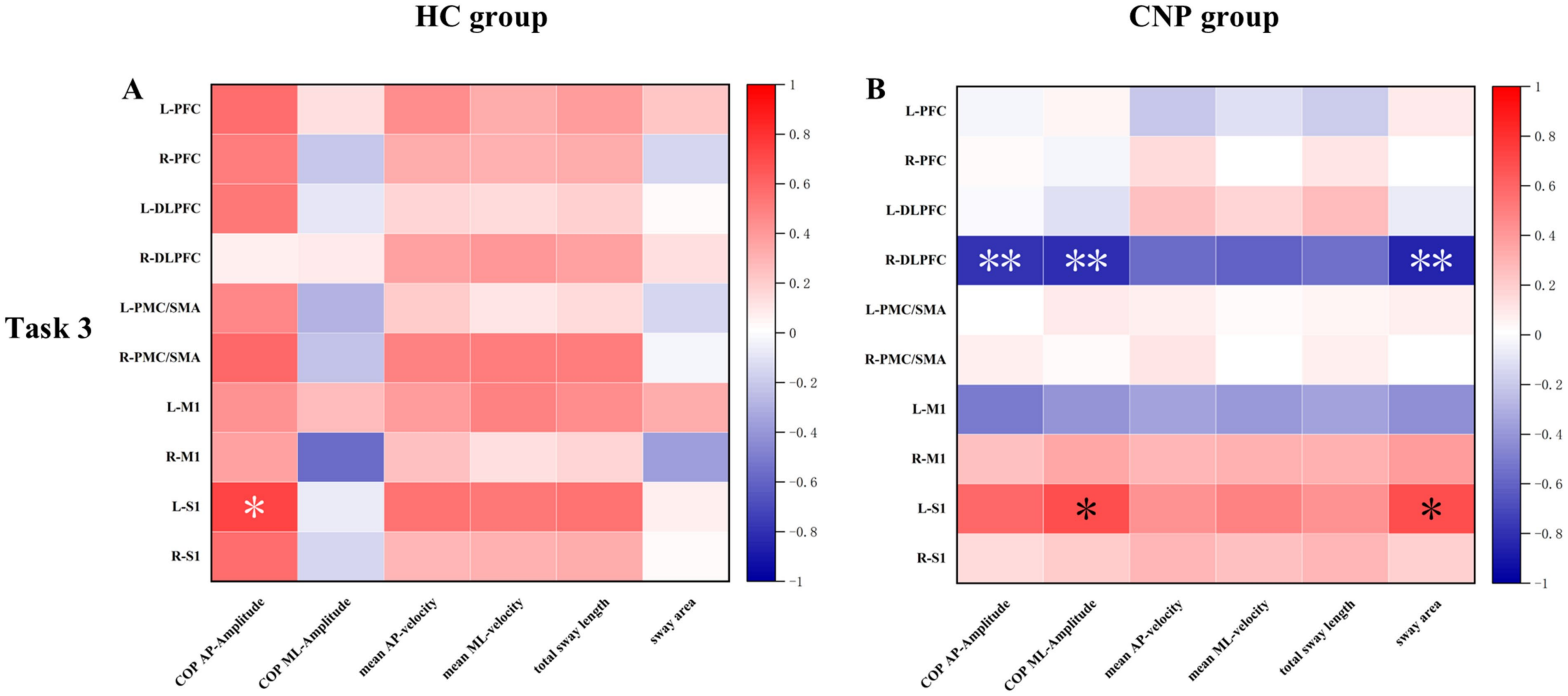
Heat map of the correlation between cerebral cortex HbO and upright standing balance parameters in the **(A)** HC and **(B)** CNP groups during task 3. L, left; R, right; PFC, prefrontal cortex; DLPFC, dorsolateral prefrontal cortex; PMC/SMA, pre-motor cortex and supplementary motor area; M1, primary motor cortex; S1, primary somatosensory cortex; ^*^ *p* < 0.05; ^**^ *p* < 0.01.

Correlation analysis revealed a significant positive correlation between activation in the left S1 and COP amplitude in the AP direction (*r* = 0.727, *p* = 0.017) in the HC group during Task 3. While in the CNP group, significant correlations were observed between the following indicators: right DLPFC activation and COP amplitude in the AP direction (*r* = −0.800, *p* = 0.005), right DLPFC activation and COP amplitude in the ML direction (*r* = −0.803, *p* = 0.005), right DLPFC activation and sway area (*r* = −0.841, *p* = 0.002), left S1 activation and COP amplitude in the ML direction (*r* = 0.689, *p* = 0.028), and left S1 activation and sway area (*r* = 0.688, *p* = 0.028) (Figure 5).

## 4 Discussion

Previous studies have demonstrated that individuals with CNP experience postural control deficits. To investigate the cortical activation for better understanding the central mechanisms underlying these deficits, balance performance and cerebral cortex activation across three different upright standing tasks were examined in this study, and the relationship between the two factors was further explored. In this study, significant differences were observed in cerebral cortex activation between the two groups across all upright standing tasks. During Task 3, the CNP group exhibited poorer balance performance in several balance parameters. A correlation between cerebral cortex activation and balance performance was observed in the CNP group, while this correlation was significantly absent in the HC group. These findings are discussed in detail below.

Postural control refers to the ability to maintain, achieve, or restore balance in any posture. This complex process involves the integration of visual, vestibular, and proprioceptive systems. The interaction among these sensory systems facilitates effective postural adjustments essential for maintaining stability in various environments (9). In this study, no significant differences were observed in balance parameters between the CNP and HC groups during the simpler tasks (Task 1 and Task 2). However, when visual input was removed, and task difficulty increased in Task 3, a significant difference was observed between the two groups. The CNP group specifically exhibited a larger COP amplitude in the AP direction than the HC group, with a large effect size of 0.282. These findings suggest that postural control deficits in individuals with CNP may not be evident during less challenging tasks, especially when visual input is available to compensate for proprioceptive deficits. Additionally, previous studies using the eyes-open Romberg test reported no significant differences in postural performance between the CNP and HC group, indicating that individuals with CNP may rely on visual cues to compensate for proprioceptive impairments (38). However, despite the removal of visual input during Task 2, no differences in balance parameters were observed between the two groups in this study. This outcome may be due to the small sample size, as the differences between COP amplitude in the ML direction (*p* = 0.052) and total sway (*p* = 0.097) between the two groups were approaching statistical significance. Furthermore, previous studies have shown increased total sway area and COP amplitude range in individuals with CNP; however, no group differences were observed in simple eyes-open static balance tasks (26, 39–41). These findings are consistent with those of previous studies, emphasizing that postural control deficits in individuals with CNP are more pronounced under conditions that challenge sensory integration.

fNIRS offers several advantages that make it particularly suitable for this study, including portability, non-invasiveness, and low sensitivity to motion artifacts during dynamic balance tasks. These features enable concurrent measurement of cerebral cortex activation during upright standing tasks. In this study, the CNP group exhibited significantly higher activation than the HC group in the left PMC/SMA and M1 during the simple Task 1, with a large effect size of 0.210 and 0.348. A previous study investigating postural control in individuals with chronic low back pain reported similar cerebral cortex activation patterns during Task 1 (21), suggesting that individuals with CNP may require additional cortical resources to manage both the demands of postural control and the interference caused by pain. These findings indicate that cerebral cortex activation may detect postural control impairments more sensitively than balance parameters measured through the force plate, as no significant differences were observed in balance parameters during Task 1. In Task 2, where visual input was blocked, the HC group exhibited significantly greater cerebral cortex activation in the left and right PFC than the CNP group, with a large effect size of 0.216 and 0.251. The PFC is involved in higher cognitive functions and plays a critical role in task adaptation and movement regulation (42). The findings suggest that the reduced PFC activation in the CNP group may contribute to the significantly larger sway area observed in these individuals. A previous study has shown that chronic pain can lead to central sensitization, resulting in an exaggerated response to non-painful stimuli (43), thereby accelerating energy depletion and manifesting as reduced cerebral cortex activation. In the more challenging Task 3 involving visual deprivation, the CNP group exhibited significantly greater activation in the right M1 than the HC group, with a large effect size of 0.304. The M1 is primarily responsible for executing fine motor movements and controlling body parts (44). As the upright standing task became more challenging, the difference in postural control between the two groups became more pronounced. Given that all participants in this study were right-handed, the left hemisphere of the brain was the dominant hemisphere (45). For the CNP group, activation of the left M1 alone was insufficient to support the task, leading to greater reliance on the right M1 activation to complete it. These findings suggest that individuals with CNP can depend on compensatory mechanisms, engaging additional cerebral cortex areas to maintain postural control, reflecting the ability of the brain to adapt and redistribute functional load (46).

In the correlation analysis, the HC group exhibited a significant positive correlation between activation in the left S1 and COP amplitude in the AP direction only during Task 3. In contrast, the activation in the left S1 exhibited a significant positive correlation with COP amplitude in the ML direction and sway area in the CNP group. Moreover, significant negative correlations between activation in the right DLPFC and both COP amplitude in the AP and ML directions, along with the sway area, were observed in the CNP group during Task 3. Previous study has shown that the S1 is primarily responsible for sensory information processing, motor coordination, and maintaining balance (47). In both the HC group and the CNP group, balance must be maintained during task 3. At the same time, according to the contralateral control principle (48), for right-handed individuals, the left hemisphere of the brain controls the movement and sensation of the right side of the body. Therefore, the results showed correlation between the left S1 and balance parameters. In the CNP group, the right DLPFC was significantly associated with balance parameters, a phenomenon that was not observed in the HC group. The phenomenon potentially occurred because individuals with CNP experienced the dual challenge of maintaining an upright standing posture while managing pain, necessitating the recruitment of broader cerebral cortex regions to support task execution. As postural control ability declined, individuals with CNP required activation of a greater area of the cerebral cortex to maintain normal balance performance during the more challenging Task 3. Specifically, the multivariate correlations between cerebral cortex activation and balance parameters in the CNP group suggested a neural compensatory response. Individuals with CNP recruited right DLPFC activation to compensate for impaired sensorimotor function.

This study has some limitations. First, neck muscle activity was not measured concurrently with the tasks, which limited the ability to assess the relationship between cerebral cortex activation, muscle activity, and postural control ability. Consequently, a deeper understanding of the central and peripheral mechanisms underlying impaired postural control in individuals with CNP was not possible. Second, a relatively small sample size was selected based on a previous study, which may have introduced selection bias. Despite this limitation, significant results were still obtained. Third, to facilitate smooth recruitment and enhance the external validity of the study, we did not impose a restriction on the duration of previous physical therapy received by individuals with CNP, which may influence the results to some extent. Finally, because the fNIRS cap probe configuration did not cover the occipital lobe, we did not include the visual cortex as a ROI. However, the presence or absence of vision was the primary difference between Task 1 and Task 2. Exploring the activation of the visual cortex would further contribute to a comprehensive understanding of brain activities in the posture control process of individuals with CNP. Further research is necessary to validate these findings and investigate additional potential indicators and neural mechanisms underlying impaired postural control in individuals with CNP.

## 5 Conclusion

Consistent with previous studies, individuals with CNP exhibited impaired postural control. The fNIRS data for the CNP and HC groups revealed that the PFC, M1, and PMC/SMA were involved in upright standing tasks. Individuals with CNP exhibited increased activation in M1 and PMC/SMA during upright stance, while activation in the PFC was reduced. Further correlation analysis revealed a neural compensatory effect during upright standing tasks in individuals with CNP. These findings expand the understanding of cerebral cortex activity, balance performance, and their relationship in individuals with CNP during upright standing tasks. The results helped to elucidate the neural mechanisms underlying postural control dysfunction in individuals with CNP. Future research should investigate whether changes in cerebral cortex activity in individuals with CNP improve following rehabilitation.

## Data Availability

The original contributions presented in the study are included in the article/Supplementary material, further inquiries can be directed to the corresponding authors.
