# Supplementary material for: Changes in cerebral cortex activation during upright standing tasks in individuals with chronic neck pain: an fNIRS study
Source: Front Neurol. 2025 Feb 28;16:1531314. doi: 10.3389/fneur.2025.1531314 (PMC11906313; doi:10.3389/fneur.2025.1531314)
Supplement: Supplementary file 1 [file Table_1.DOCX]

**Appendix Table 1**. Results of the one-way ANOVA for HbO concentration during upright standing tasks between the groups

| **ROIs** | | **HC group (n=10) vs CNP group (n=10)** | | |
| --- | --- | --- | --- | --- |
|  |  | **F** | ***P*** | **η^2^** |
| **Task 1** |  |  |  |  |
| Left PFC | | 0.944 | 0.344 | 0.050 |
| Right PFC | | 0.615 | 0.123 | 0.127 |
| Left DLPFC | | 0.396 | 0.537 | 0.022 |
| Right DLPFC | | 0.343 | 0.566 | 0.019 |
| Left PMC/SMA | | 4.788 | **0.042** | 0.210 |
| Right PMC/SMA | | 0.266 | 0.612 | 0.015 |
| Left M1 | | 9.598 | **0.006** | 0.348 |
| Right M1 | | 1.495 | 0.237 | 0.077 |
| Left S1 | | 4.042 | 0.060 | 0.183 |
| Right S1 | | 0.865 | 0.365 | 0.046 |
| **Task 2** |  |  |  |  |
| Left PFC | | 4.952 | **0.039** | 0.216 |
| Right PFC | | 6.035 | **0.024** | 0.251 |
| Left DLPFC | | 2.876 | 0.107 | 0.138 |
| Right DLPFC | | 3.117 | 0.094 | 0.148 |
| Left PMC/SMA | | 2.795 | 0.112 | 0.134 |
| Right PMC/SMA | | 1.542 | 0.230 | 0.079 |
| Left M1 | | 0.254 | 0.621 | 0.014 |
| Right M1 | | 0.068 | 0.797 | 0.004 |
| Left S1 | | 2.710 | 0.117 | 0.131 |
| Right S1 | | 0.009 | 0.927 | 0.000 |
| **Task 3** |  |  |  |  |
| Left PFC | | 0.629 | 0.438 | 0.034 |
| Right PFC | | 1.202 | 0.287 | 0.063 |
| Left DLPFC | | 0.678 | 0.421 | 0.036 |
| Right DLPFC | | 0.622 | 0.441 | 0.033 |
| Left PMC/SMA | | 1.006 | 0.329 | 0.053 |
| Right PMC/SMA | | 1.378 | 0.256 | 0.071 |
| Left M1 | | 0.525 | 0.478 | 0.028 |
| Right M1 | | 7.873 | **0.012** | 0.304 |
| Left S1 | | 0.011 | 0.919 | 0.001 |
| Right S1 | | 0.010 | 0.920 | 0.001 |

Task 1: standing on the force plate with eyes open and both feet; Task 2: standing on the force plate with eyes closed and both feet; Task 3: standing on the force plate with eyes closed and one foot. PFC, prefrontal cortex; DLPFC, dorsolateral prefrontal cortex; PMC/SMA, pre-motor cortex and supplementary motor area; M1, primary motor cortex; S1, primary somatosensory cortex.
